# Mantle cell lymphoma treatment options for elderly/unfit patients: A systematic review

**DOI:** 10.1002/jha2.311

**Published:** 2021-11-09

**Authors:** Tahera Alnassfan, Megan J. Cox‐Pridmore, Azzam Taktak, Kathleen J Till

**Affiliations:** ^1^ Department of Molecular and Clinical Cancer Medicine University of Liverpool Liverpool UK; ^2^ Medical Physics and Clinical Engineering Royal Liverpool University Hospital Liverpool UK; ^3^ Authors Tahera Alnassfan and Megan J. Cox‐Pridmore contributed equally to the review

**Keywords:** elderly, ibrutinib, MCL, R‐CHOP, treatment

## Abstract

Mantle cell lymphoma (MCL) is a rare B‐cell non‐Hodgkin lymphoma (NHL) that is aggressive and incurable with existing therapies, presenting a significant unmet clinical need. MCL occurs mainly in elderly patients with comorbidities; thus, intense treatment options including allogeneic stem cell transplantation (Allo‐SCT) are not feasible. New treatment options are emerging for this elderly/unfit treatment group, we therefore conducted a systematic review to determine whether they offered an advance on the existing recommended treatment, R‐CHOP. The search strategies to identify MCL therapies were designed to capture the most relevant studies from 2013 to 2020. Following preferred reporting items for systematic reviews and meta‐analyses and population,interventions, observations and study design analysis, R‐CHOP, ibrutinib and bendamustine plus rituximab (BR) were taken forward for critical and statistical analysis. All three therapies were effective in increasing the overall survival (OS) and progression‐free survival of elderly/unfit patients with MCL. However, none resulted in a significant increase in OS compared to R‐CHOP. In addition, R‐CHOP had a better toxicity profile when compared to both ibrutinib and BR. We therefore conclude that treatment of elderly/unfit patients with MCL is still a significant unmet clinical need; and suggest that outside of the clinical trial setting, R‐CHOP should remain the recommended front‐line treatment for this patient group.

## 1 INTRODUCTION

Mantle cell lymphoma (MCL) was first defined as an aggressive subtype of non‐Hodgkin's (NHL) B‐cell lymphoma in 1970 and accounts for around 3% to 6% of B‐cell NHLs [1] and is more prevalent in males than females (4:1). Despite being classified as a distinct entity for over 50 years, MCL remains difficult to treat [2]. The MCL‐International Prognostic Index (MIPI) is used to define the prognosis of MCL patients [3]. According to the MIPI, patients can be categorised as low risk with a median overall survival (OS) rate of 5 years, intermediate risk with OS of 51 months and high‐risk group shows 29 months OS rate [1].

MCL is derived from the B‐cells in the mantle region of secondary follicles of lymph nodes [4], and is classically defined by the presence of a pathognomonic chromosomal translocation t(11;14)(q13;q32) [5]. This translocation results in the fusion of the CCDN1 gene‐encoding cyclin D1 to the immunoglobulin heavy chain promoter, leading to the overexpression of cyclin D1 [6]. The biological functions of cyclin D1 include regulation of transcription, induction of chromosomal instability and modulation of epigenetic mechanisms [2]. However, not all MCL cells express cyclin D1 [7]. This has led to the identification of other genetic factors that may be of importance in MCL such as the transcription factor SOX11 which is expressed in approximately 90% of MCL cases; and can be used as a useful diagnostic marker to identify both cyclin D1‐positive and D1‐negative MCL [6, 7]. Based on the clinical presentation and molecular composition, MCL has been divided into two subtypes; nodal patients who have lymphadenopathy and non‐nodal MCL who do not have enlarged lymph nodes [8]. Nodal MCL is the most common variant comprising approximately 80% of cases. The malignant lymphocytes from this subtype have overexpression of SOX11 and an un‐mutated *IGHV* genotype [9]. Although lymphadenopathy, splenomegaly and gastrointestinal infiltration are the most prevalent symptoms in patients with MCL [6], 10–20% of patients present without these clinical features. The malignant lymphocytes in these non‐nodal MCLs do not express SOX11 and exhibit hypermutated *IGHV* genotype [9]. Taken together, the addition of cyclin D1, SOX11, and *IGHV* analysis has broadened criteria for the accurate diagnosis of MCL which is useful in the selection of the most appropriate treatment. In addition, MCL B cells are defined by expression of CD5, CD19, CD20 and CD22; and the surface immunoglobulins IgM and IgD.

This heterogeneity of MCL, together with the fact that patients respond poorly to therapy, means that a variety of different therapeutic treatments have been trialled. These vary in toxicity, disease targets and mechanism (Table 1). For fit and healthy individuals, allogeneic stem cell transplantation (allo‐SCT) is routine [10]. More intense drug regimens such as maxi‐R‐CHOP, involving cytarabine, have also resulted positive therapy responses in young‐fit patients [10]. However, the MCL population is mostly over the age of 68 and generally cannot tolerate intense therapeutic regimens or transplantation [2].

**TABLE 1:** Current therapies for MCL treatment. Demonstrating their classification and US Food and Drugs Administration (FDA) approval status [4, 11–20, 21–24]

Therapeutic classification	Treatment options	US Food and Drugs Administration (FDA) approval year
**Inhibitors**	Proteasome inhibitor Bortezomib	2006 [25]
	Bruton's tyrosine kinase inhibitors Ibrutinib Acalabrutinib Zanubrutinib	Ibrutinib – 2013 [26] Acalabrutinib – 2017 [11] Zanubrutinib – 2019 [12]
**Immunotherapy**	Rituximab	1997 [27]
	Lenalidomide (analogue of thalidomide)	2013 [28]
**Chemotherapy**	Bendamustine	2008 [15]
	Chlorambucil	Still undergoing clinical trials. [29]
**Chemo‐immunotherapy**	R‐CHOP (rituximab, cyclophosphamide, doxorubicin, vincristine, prednisone) Variations of R‐CHOP VR‐CAP (bortezomib, rituximab, cyclophosphamide, doxorubicin, prednisone Maxi‐R‐CHOP (higher CHOP doses, followed by cytarabine and autologous stem cell transplant. R‐hyperCVAD (rituximab, cyclophosphamide, vincristine, doxorubicin, dexamethasone) VcR‐CVAD (bortezomib, rituximab, cyclophosphamide, vincristine, doxorubicin, dexamethasone)	R‐CHOP – Addition of rituximab to CHOP therapy. [30] VR‐CAP – Phase III trials. [31] Maxi‐R‐CHOP – Current R‐CHOP regime with added high doses of cytarabine – Phase II trial. [16] R‐hyperCVAD – Currently still undergoing clinical trials. [32] VcR‐CVAD – Currently still undergoing clinical trials. [33]
	BR (bendamustine, rituximab)	BR – Phase III trials. *Trial has been completed, awaiting FDA approval. [34]
	Cytarabine‐based induction	Cytarabine‐based induction – Phase III trials. [7]

R‐CHOP (rituximab, cyclophosphamide, doxorubicin, vincristine, prednisolone) is the current recommended treatment for individuals ineligible for intense treatments [22]. It is reasonably tolerated, in comparison to other therapeutics. The various components of CHOP contribute to its effectiveness in different ways; but all result in an inhibition of cell division and/or cell death. The major mechanism of action of cyclophosphamide (C) is due to its hydroperoxide metabolite, 4‐hydroperoxycyclophosphamide (4‐HC), an interstrand DNA cross‐linking agents which leads to DNA damage [35]. Doxorubicin (H) also found to play a key role in DNA damage by inserting itself between DNA bases which results in cell death [36]. Vincristine (O), on the other hand, binds to the protein tubulin and inhibits cell duplication; and prednisolone (P) is a corticosteroid [37]. The addition of rituximab, an anti‐CD20 antibody, to the previous standard treatment, CHOP, enabled an increased response and OS [27].

**FIGURE 1:**
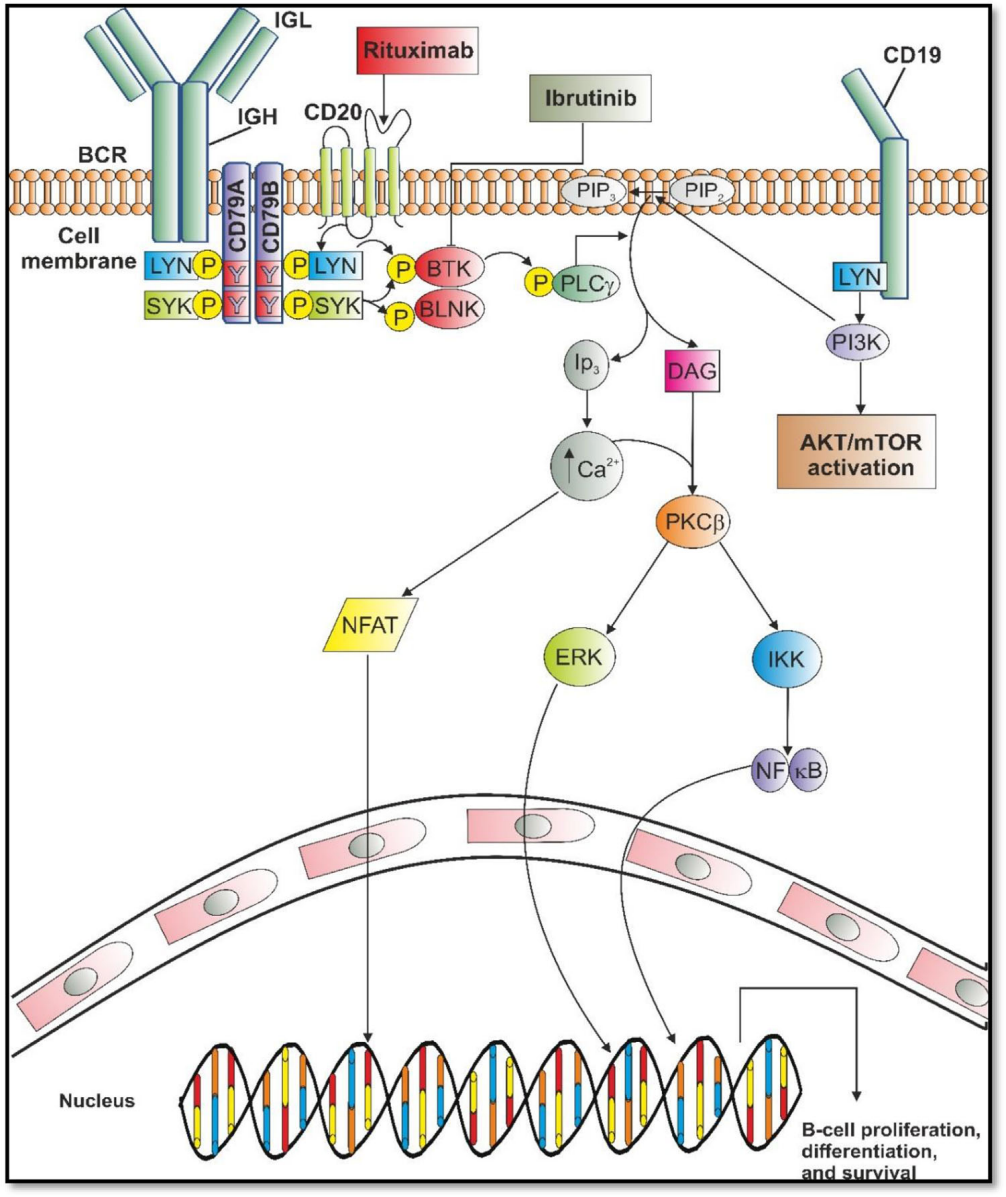
Mechanisms of action of rituximab and ibrutinib.

The human CD20 protein is a membrane‐embedded molecule express on the surface of B cells including those of MCL [1]. To date, its role in B‐cell receptor (BCR) signalling is not fully understood [38]. Despite its function not being fully elucidated, the expression of CD20 on B‐NHL cells led to the molecule being targeted as a therapeutic approach (Figure 1 [19]). Rituximab is a chimeric monoclonal antibody which binds CD20 expressing cells [39], and is thought to act by inducing antibody‐dependent cellular cytotoxicity (ADCC) within the malignant B cells [40]. Natural killer (NK) cells produce IFNγ, when in contact with the CD20 positive cells that have bound with rituximab. Both direct apoptotic and indirect mechanisms involving constituent immune effector cells can contribute to ADCC [41, 42]. Rituximab can, consequently, be a successful treatment against MCL. However, as with all therapeutics, R‐CHOP is linked with toxicities including peripheral neuropathy, myelosuppression and cardiac toxicities further restricting treatment in the elderly/infirm treatment group with their multiple comorbidities [43]. Therefore, other front‐line treatment options are required.

The alkylating agent, bendamustine, has been used in combination with rituximab as an alternative approach, and has displayed promising antineoplastic effects, resulting in the potential for bendamustine‐rituximab (BR) therapy to be used as an alternative first‐line treatment to R‐CHOP [44]. Despite the fact that the precise mechanism of action of bendamustine is still poorly understood, it is known to cause significant DNA damage [45]. However, secondary mechanisms of action are also thought to contribute, these include: (1) ineffective DNA repair, (2) suppression of p53‐dependent DNA‐damage stress response and (3) suppression of mitotic checkpoints; all of these events contribute to mitotic catastrophe and apoptosis [46].

Although R‐CHOP significantly improves initial symptoms and life expectancy, treatment failure is inevitable leading to relapsed or refractory MCL [18, 47]. Consequently, a second approach is required. The most frequent second‐line option is to target the BCR pathway with ibrutinib, a Bruton's tyrosine kinase (BTK) inhibitor (Figure 1 [8, 20]).

The BCR pathway plays an important role in normal and neoplastic B cells. In normal B cells, the BCR signalling pathway is initiated via antigen binding to surface immunoglobulin resulting in the phosphorylation of CD79A and CD79B [14]. This leads to the phosphorylation of BTK and phosphatidylinositide‐3‐kinase‐δ (PI3Kδ) and activation of downstream signal pathways involving mitogen‐activated protein kinase (MAPK), mammalian target of rapamycin (AKT/mTOR), nuclear factor of activated T cells (NFAT) and nuclear factor kappa B (NF‐*κ*B) [47]. BTK‐mediated signalling is involved the growth, motility adhesion and proliferation of both healthy and malignant B cells [48].

With regard to MCL, the malignant lymphocytes have constitutive BCR signalling which is crucial to the pathogenesis of the disease, and as a result, BTK signalling is unregulated [10]. Ibrutinib binds to BTK, blocking its phosphorylation and thereby downstream signalling events (Figure 1 [10]). After administration ibrutinib is metabolised, CYP3A and CYPRD6, its active metabolite, then forms an irreversible covalent bond to the cysteine residue 481 on the BTK molecules, altering modification of tyrosine 223 on exon 8 [17, 49]. This interrupts BCR signalling, and disrupts the MCL cell survival and disease progression. The BTK^C481S^ mutation has been shown to lead to resistance and treatment failure in CLL; however, this mutation does not play a role in the primary or acquired resistance to ibrutinib which is seen in MCL patients [21]. Ibrutinib has also been shown to inhibit off‐target kinases, which may result in the toxicity that limits its overall clinical benefit [20].

Following ibrutinib treatment, initial response rates are favourable in most patients. However, all participants ultimately experience resistance to treatment within an average of 6–10 months [17]. One of the mechanisms of resistance is kinome‐adaptive reprogramming [5, 10]. This leads to the activation of the PI3K/AKT/mTOR pathway and integrin‐β1 signalling and results in proliferation and increased adhesion of MCL stromal cells [10]. Adhesion within the stromal micro‐environment mediated by the integrin α4β1 and the chemokine receptors CXCR4 and CXCR5 has been demonstrated to be involved in drug resistance [50]. In addition, integrins and chemokines play an important role in the pathogenesis of MCL and are responsible for directing and maintaining the malignant MCL cells in a permissive micro‐environmental niche within lymphoid tissues [51]. Another signalling pathway up‐regulated in resistant MCL cells is that of the transcription factor NF‐*κ*B pathway. The NF‐*κ*B pathway also promotes the cell growth and survival of MCL cells. This protection is mediated, in part, through the up‐regulation of tumorigenic cytokines [21, 50].

In conclusion, MCL is highly aggressive, incurable form of NHL. Whilst current treatments partially limit tumorigenesis and suppress disease symptoms, the duration of remission is short and all patients eventually relapse [2, 21, 52]. The aim of this systematic review is to identify which of the currently available therapeutics provide the best option for treating elderly and unfit patients; taking into account both survival benefit and the toxicities of the drugs – an important factor when considering this patient group. A systematic review of the available data enables us to identify the best treatment options for patients suffering from MCL that cannot endure intense treatment or allogeneic stem cell transplant (allo‐SCT).

## 2 METHODS

### 2.1 Criteria for search

This systematic review was formulated through specific inclusion criteria to identify therapeutic regimens used to treat elderly or unfit MCL patients (hereafter referred to as elderly/unfit). The analysis was performed via the electronic database PubMed, with a limit for extraction between the years 2013 and 2020. The start date was chosen as the tyrosine kinase inhibitor ibrutinib, a promising new drug for the treatment of elderly/unfit MCL was first in used in 2013 [53]. Publications were identified using a combination of search terms covering a broad area in order to ensure that all publications relevant to the research question were identified. These included: ‘Mantle cell lymphoma treatment’; ‘Mantle cell lymphoma therapy’ and ‘B‐cell non‐Hodgkin's lymphoma, including mantle cell lymphoma’. Nine hundred and one articles were identified using these terms; the articles had contrasting study designs, including retrospective, randomised, multicentre, observational, cohort, real world, open‐label and prospective.

**FIGURE 2:**
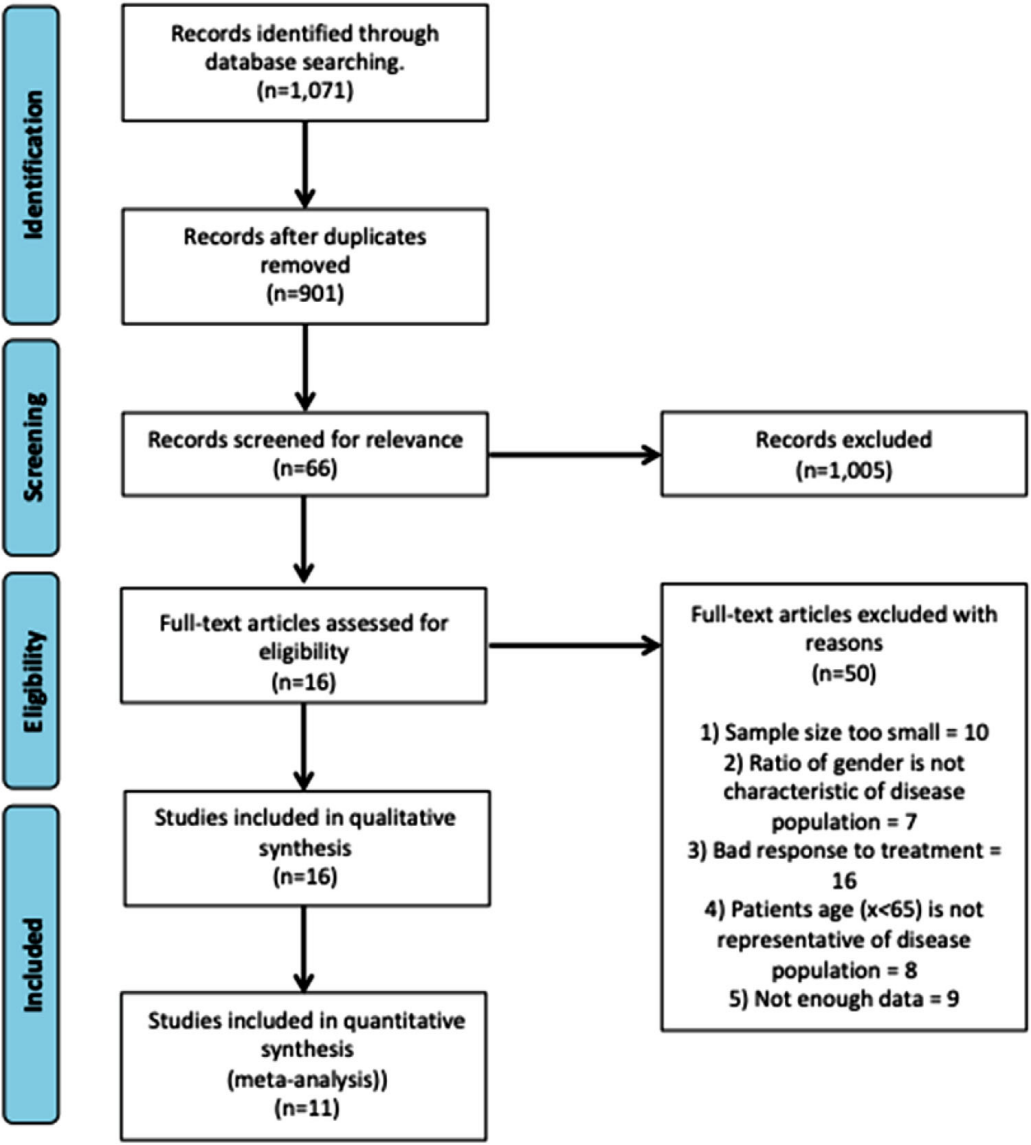
Selection criteria for systematic review. Papers were screened according to PRISMA. PIOS was then used to exclude papers with insufficient data and those which did not represent the patient demographics

The identified papers were therefore screened according to the preferred reporting items for systematic reviews and meta‐analyses (PRISMA). Papers were then analysed on population, interventions, observations and study design (PIOS) to remove papers which had insufficient data or were not representative of the disease demographics. Phase III studies were included within these criteria if they were in a clinical setting and the treatments had subsequently been approved by the Food and Drug Administration (FDA). This screening enabled inclusion of all treatments in current clinical practice outside of the trial setting. The detected articles were extracted for duplicates and further analysed through title and abstract screening. The final papers underwent additional inspection with regard to the exclusion criteria (Figure 2).

With regard to inclusion criteria, since not all unfit patients are elderly, papers regarding unfit subjects under the age of 65 were incorporated if the treatment was well tolerated. As MCL is also more prevalent in males than females (a 4:1 ratio), studies that did not reflect this bias were also excluded to ensure that the studies taken forward for analysis accurately represented the patient the population. MCL is a relatively rare lymphoma, and therefore large patient databases were not always available. However, studies with small sample size (*n* < 10) were removed to minimise the incorporation of bias. With regard to therapy inclusion criteria, papers which focused on transplantation were excluded because elderly/unfit individuals are not able to tolerate the intense treatment regimens required prior to allo‐SCT. Finally, papers which looked at B‐NHL treatment including MCL were removed, if the data did not include separate data on MCL patients.

### 2.2 Data extraction and analysis

Data extraction was performed from the finalised list of papers. Study design, sample size, study setting, average length of follow‐up, participant characteristics (age, gender, stage) and outcomes/findings regarding OS, progression‐free survival (PFS) and adverse events (AEs) were extracted. Due to the variation in reporting methods for age, OS, PFS and AEs we were unable to perform meta‐analysis on this data; therefore, qualitative assessment was performed. However, we were able to perform sufficient data extraction to perform meta‐analysis on patient stage and gender (percentage of males). This enabled us to assess whether or not the different treatment regimens we examined were used to treat patients with similar characteristics.

### 2.3 Statistical analysis

Meta‐analysis of patient stage and gender was performed by plotting the percentages reported for each study with error bars calculated assuming binomial distribution of the data as:

$$1.96+\surd \left(p*\left(1-p\right)/n\right),$$

where *p* is the percentage of males or stage and *n* is the sample size in the study.

For analysis between treatment groups, firstly, the harmonic mean and harmonic standard error were calculated for each group as follows:

$$\mathrm{Harmonic}\;\mathrm{Mean}\;=\sum_{i}\omega_{i}\times Mean_{i}.\;$$

The ω’s are proportional to the sample size and add up to 1

$$\mathrm{Harmonic}\;\mathrm{Standard}\;\mathrm{Error}\;=\sqrt{\frac{1}{\sum_{i}\eta_{i}}},\;$$

where $\eta_{i}=\frac{1}{s.e{._{i}}^{2}}$.

## 3 RESULTS

### 3.1 Description of included studies

The PRISMA diagram for selection of studies in shown in Figure 2. The original total of records from the PUBMED database search was 1071 articles. Following removal of duplicate papers, 901 papers remained; of these 805 articles were removed after the titles and abstracts were screened, leaving 66 articles for full screening. From these papers, 16 articles met all the inclusion criteria, and were taken forward for qualitative synthesis (Table 2 [13, 23, 24, 32, 54, 55, 56, 57, 58, 59–61, 63, 64, 65, 67]).

**TABLE 2:** Data extraction table of all articles used in the systematic review

Study(author/date)	Design	Samplesize	Study setting	Mediumfollow‐up	Participantcharacteristics	Outcomes/findings	Adverse Events ≥stage 3/ Treatment discontinuation
**R‐CHOP**							
Verhoef et al [65] [Paper 1]	Phase III Lym‐3002 study	*n* = 487	128 centres in 28 countries	5 years	Age = 65 years (26–88) (VR‐CAP), 66 years (34–82) (R‐CHOP) Males = 73% (VR‐CAP), 73% (R‐CHOP) Stage III/IV = 100%	Longer duration and quality of response with VR‐CAP verses R‐CHOP. PFS 24.7 months (VR‐CAP), 14.4 months (R‐CHOP). VR‐CAP had higher rates of toxicity. More evident in low/intermediate risk MIPI	Thrombocytopenia (6%), neutropenia (67%), leukopenia (29%), lymphocytopenia (9%), infections/infestations (14%).
Kang et al [56] [Paper 2]	Retrospective review	*n* = 131	15 Medical centres in Korea	20 m	Age = 63 years (26‐78) Males = 78% Stage III/IV = 80%	Addition of rituximab did not significantly affect therapeutic outcome. OS at 2 years was 67%.	
Das Ch et al [55] [Paper 8]	Retrospective analysis	*n* = 51	North Indian tertiary care centre	20.7 m	Age = 57 years Males = 80% Stage III/IV = 80%	The addition of rituximab to current treatments showed increased response rate and PFS. PFS 51% (Rituximab‐containing treatment) compared to 27%. OS was 78% compared to 72%.	
Jeon et al [24] [Paper 15]	Retrospective analysis	*n* = 70	Catholic Haematology Hospital, Seoul	11 years	Age = 64 years (26–84) Males = 81% Stage III/IV = 91%	R‐CHOP showed better results in early metabolic responders than delayed responders. Five‐year OS 7.84 months, PFS 3.34 month.	
**R‐CHOP/BR**							
Villa *et al* [33] [Paper 3]	Retrospective population‐based analysis	*n* = 101	Patients in British Columbia compared to historical cohort	8 years	Age = 73 years (63‐90) (BR), 72 years (63‐87) (R‐CHOP) Males = 65% (BR), 70% (R‐CHOP) Stage III/IV = 91% (BR), 93% (R‐CHOP)	BR had significant improvements in PFS but not OS compared to R‐CHOP. PFS 56% (BR), 35% (R‐CHOP). OS 64% (BR), 55% (R‐CHOP). However, results are suboptimal within individuals of high risk.	Significant non‐relapse‐related fatal adverse event rate in the BR cohort.
**Study** **(author/date)**	**Design**	**Sample** **size**	**Study setting**	**Medium length of follow‐up**	**Participant** **characteristics**	**Outcomes/findings**	**Adverse Events ≥** **stage 3/ Treatment discontinuation**
Okay et al [60] [Paper 4]	Retrospective, multicentre study	*n* = 78	Two reference haematology departments in Turkey	3 years	Age = 62 years (34–86) Males = 78% Stage III/IV = 91%	R‐CHOP and BR had average OS 77.8 months, DFS 20.6 months. MIPI and neutrophil count affected OS (*p* = 0.047) and (*p* = 0.001), respectively. BR preferred salvage treatment. Ara‐C not favourable in elderly	
[61] [Paper 5]	Retrospective review study	*n* = 70	Six Australian tertiary centres	37 m	Age = 69 years (60–91) Males = 74% Stage III/IV = 94%	Ara‐C‐containing therapy, and BR had improved OS and PFS results compared to R‐CHOP. OS R‐CHOP 3.9y, Ara‐C 67 months (54–81); PFS R‐CHOP 42 m, Ara‐C 45 m (35–55), BR 35.4 m. HyperCVAD associated with increased hospitalization.	R‐CHOP 48% haemic. 87% of patients. BR Reduced dose 49%; 15% withdrew.
[13] [Paper 13]	Randomised, multi‐centre, phase III study	*n* = 447	Clinical centres located in Canada, United States, Brazil, Peru, Mexico, Australia and New Zealand	5 years	Age = 60 years (BR), 58 years (R‐CHOP) Males = 61% (BR), 59% (R‐CHOP) Stage III/IV = 45% (BR), 45% (R‐CHOP)	Overall response rate was higher in BR than R‐CHOP, 97% AND 91% respectively. However, there was a higher incident of adverse reactions with BR.	BR – 4% withdrew due to AEs
**R‐ CHOP ± Ibrutinib**							
Kumar et al [35] [Paper 16]	Retrospective chart review	*n* = 386	Memorial Sloan Kettering Cancer Centre	74 m	Age = 64 years (28–90) Males = 76% Stage III/IV = 88%	First line of treatment OS, 9.7 years, PFS 4.0 years. Second line of treatment OS 41.1 months, PSF 14.0 months. Progressive shortenings to response and survival at each line of treatment	Ibrutinib ‐ 25.6% treatment interruption due to toxicity – most common atrial fibrillation followed by bleeding.
**Ibrutinib**							
Broccoli et al [54] [Paper 9]	Observational, retrospective, multi‐centre study	*n* = 77	29 Italian centres	36 m	Age = 65 years (35–81) Males = 77% Stage III/IV = 95%	OS 16 months, PFS 12.9 months.	
**Study** **(author/date)**	**Design**	**Sample** **size**	**Study setting**	**Medium length of follow‐up**	**Participant** **characteristics**	**Outcomes/findings**	**Adverse Events ≥** **stage 3/ Treatment discontinuation**
O'Brien et al [59] [Paper 10]	4 phase III randomised controlled studies	*n* = 278	UK based clinical trial	16 m	Age = 67 years (30–89) Males = 67%	Adverse effects more common with Ibrutinib than temsirolimus but similar. PFS 15.6 months (Ibr), 6.2 months (tem). OS 30.3 months (Ibr), 23.5 months (tem).	36% haemic (6% atrial fibrillation; 3% diarrhoea) – 6% death; 12% discontinued due to AEs.
Jeon et al [24] [Paper 14]	Observational, retrospective, cohort study	n = 33	Catholic Haematology Hospital, Seoul	2 years	Age = 65 years (40–79) Males = 80% Stage III/IV = 58%	Favourable OS and PFS with Ibrutinib, 35.1 months and 27.4 months, respectively. Failure of treatment led to inferior survival outcomes.	6.1% atrial fibrillation; 9% discontinued due to drug related complications
Sharmen et al (2020) [Paper 17]	Retrospective chart review	*n* = 159	US Oncology Network – Electronic medical records database	16 m	Age = 71 years Males = 76% Stage III/IV = 87%	Ibrutinib showed high toxicity and adverse events. OS 25.8 months, PFS 19.5 months.	9.4% atrial fibrillation; 11.3% diarrhoea
**Other**							
Chang et al [32] [Paper 6]	Prospective study	*n* = 15	Wisconsin Oncology Network (WON)	7.8 years	Age = 61 years Males = 80% Stage III/IV = 100%	VcR‐CVAD at 6 years had PFS 53% which was not affected by MIPI and OS 70%. Maintenance rituximab contributed to results. Acute toxicities were observed.	
Rule et al [62] [Paper 7]	Randomised, open‐label, multi‐centre study	*n* = 370	UK National Cancer Research Institute	6 years	Age = 66 years (60–91) (F/C), 66 years (36–88) (F/C/R) Males = 79% (F/C), 74% (F/C/R) Stage III/IV = 90% (F/C), 85% (F/C/R)	Addition of rituximab to fludarabine and cyclophosphamide significantly improved patient outcome, but with late toxicity. PFS improved from 14.9 to 29.8 months. OS from 37.0 to 44.5 months	
Smith et al [61] [Paper 12]	Contemporary real‐world observational study	*n* = 335	Established population cohort	2 years	Age = 74 years (35–96) Males = 67% Stage III/IV = 94%	OS 91.1% (Rituximab‐containing treatment), 76.8% (non‐rituximab‐containing treatment).	1.5% withdrew due to AEs

Eight papers involved the use of chemo‐immunotherapy in these papers R‐CHOP were compared with BR (four papers), VR‐CAP (one paper) or Aca‐C (one paper). For one of the papers which compared R‐CHOP with BR, there was no data for age or percentage of males for the latter, and the data on BR therefore were not included in subsequent analysis as it did not meet the PIOS criteria. In addition, VR‐CAP and Ara‐C were not tolerated in elderly patients, so data on these treatments did not form part of our subsequent analysis. Five papers examined kinase inhibitor therapy using ibrutinib.

The main aim of this systematic review was to compare the efficacy of the different treatment regimens used for unfit/elderly patients with MCL; however, there was insufficient data to perform qualitative analysis of the survival data (Table 2). Therefore, qualitative assessment was used to assess the efficacy of the current treatment options available for elderly/unfit patients with MCL. Of these papers, 11 had sufficient data on stage and gender to enable quantitative meta‐analysis to be performed (Table 2).

### 3.2 Description of study characteristics

**FIGURE 3:**
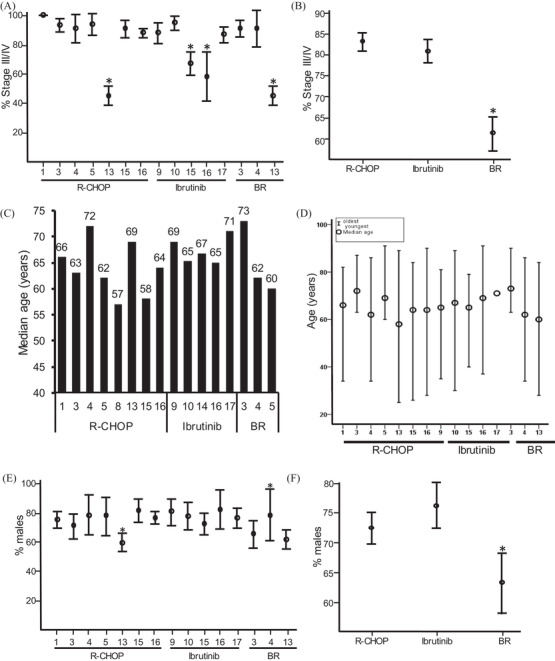
Patient demographics. Stage of disease (A) data from individual papers and (B) pooled data. Age of patient group (C) average age and (D) age range. % males; (E) data from individual papers and (F) pooled data. Ranges in (A), (B), (D) and (F) assume a binomial distribution; in (D), the actual age range is used. Circles represent the median values (B). * *p* < 0.05

To analyse and compare the data regarding the different treatment regimens, we began by analysing whether, or not, the characteristics of the patients used in the different studies were the same. Firstly, we analysed the stage of the patients entered in each of the studies. The majority of the patients were in stage III/IV across all the data sets (Figure 3A). However, a lower percentage of cases in stage III/IV was seen in the studies undertaken in papers 13, 15 and 10 [13, 24, 59]. In addition, when comparing between the therapies, the stage of patients treated with BR was significantly lower than that of those treated with R‐CHOP or ibrutinib (Figure 3B).

We next analysed the average age of the patients in each of the studies (Figure 3C). The age range of the patients varied between the different studies; some concentrated on elderly patients (papers 3 & 5 [33, 61]), whereas the majority of the studies included both elderly and unfit patients; there was no age range data for study 17 [63]. However, the median age of the patients from each of the studies was in their 60s apart from paper 13 where the mean age was late 50s, and papers 3, 16 and 17 which were early 70s [33, 63, 66] (Figure 3D).

Finally, we compared the percentage of males in each study; MCL has a male to female bias of 3–4:1. We therefore compared the percentage of males in each study taking into account the sample size. The percentage of males was comparable across most of the studies (Figure 3D), apart from paper 13 [13] where the number was significantly lower than the reported ratio, and paper 14 [24] where it was significantly higher. No age range was reported for study 17 [63]. We then compared the percentage of males between the different therapies (Figure 3E), and this indicated that the studies involving BR had a significantly lower percentage of males than those using R‐CHOP or ibrutinib.

Taken together, these data indicate that the patient characteristics of BR patients were significantly different from patients treated with R‐CHOP or ibrutinib with regard to stage and the proportion of males. The fact that there are less patients with stage III/IV is particularly important in analysing the outcome data, as high stage predicts a less favourable response to therapy [52].

### 3.3 Description of treatment responses

The efficacy of any treatment is determined by two metrics, survival data and toxicity of the treatment; the latter will be described in the next section. With regard to survival, PFS and OS were assessed in most of the studies, in some this was reported as % of patients in each group at a certain time point, and in others as the average number of months following treatment (Table 2). There was insufficient data to analyse the OS between the different treatment regimens; however, PFS is a good indicator of the efficacy of the treatment [67]. We therefore compared the PFS of the different treatment regimens.

**FIGURE 4:**
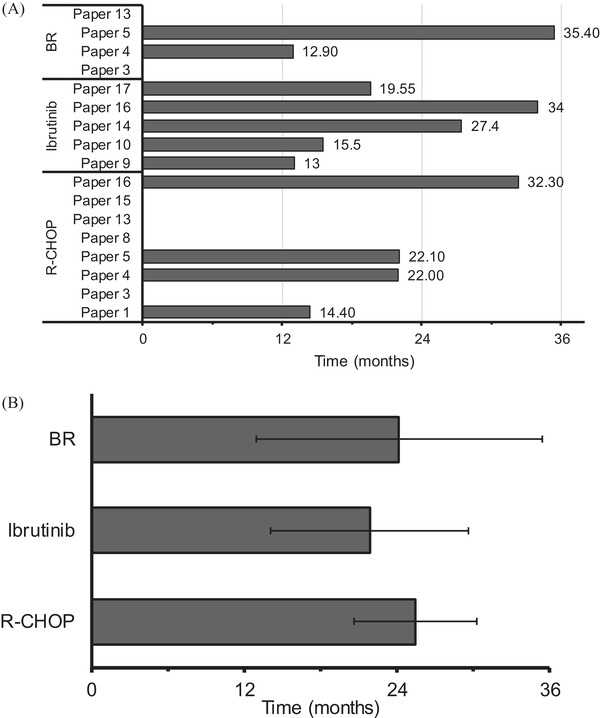
Progression free survival: (A) Data from individual papers and (B) pooled data

Data on the number of months of PFS were available for 4/8 patients treated with R‐CHOP, 2/4 with BR and all 5 studies with ibrutinib. The range of PFS between the different studies was similar for all treatments; R‐CHOP 14–32 m; ibrutinib 13–27 m; BR 13–35 m (Figure 4A). The similarity of the survival ranges for the three treatments was reflected by the fact that when the average PFS for each treatment was calculated, the PFS for R‐CHOP, BR and ibrutinib was approximately 2 years (Figure 4B).

### 3.4 Description of side effects

With regard to the tolerability of the different treatments, both the severity of the AEs and the number of patients who discontinued treatment due to side effects contribute to this metric. Data on patients who had serious (≥ grade 3) toxicities were reported in 10 of the studies (Table 2). The most common serious AEs with all treatments were haemic; these appeared to be more severe in patients treated with BR. Of note was the fact that between 6% and 9% of patients treated with ibrutinib suffered from atrial fibrillation (≥ grade 3), which in some cases led to death. The discontinuation rate due to toxicity was high with both ibrutinib (≤25%) [24, 63] and with BR≤16% [61]; no data were reported for R‐CHOP.

## 4 DISCUSSION

To our knowledge, this is the first systematic review of treatment options for elderly/unfit patients with MCL. Our study identified five different treatment regimens which had been examined within the criteria of this review: elderly/unfit patients after the introduction of ibrutinib in 2012. The three most frequently studied treatments were R‐CHOP (eight studies [13, 23, 33, 55, 56, 60, 61, 65]), ibrutinib (five studies [24, 54, 59, 63]) and bendamustine (three studies [13, 33, 60]). We found that treatment outcomes in patients treated with R‐CHOP were not inferior to those with ibrutinib or BR.

The strength of this study is that the data we analysed were unselected, obtained different clinical settings, and in a number of different countries. Although our data included randomised clinical trial data in both single and multi‐centre studies, as well as retrospective analysis, this was true for all the treatments systematically reviewed. Moreover, the outcome data were broadly similar regardless of the setting, or continent, where it was acquired. Therefore, we believe that our data give a real‐world perspective on the efficacy of the treatment choices for elderly patients with MCL.

R‐CHOP is the currently approved front‐line treatment for elderly unfit patients outside of the clinical trial setting [7]. Therefore, several studies compared the efficacy of new treatment regimens with R‐CHOP. All three studies using BR compared the treatment in randomised control studies with R‐CHOP. While only one study with ibrutinib compared it with R‐CHOP, this was because the studies with ibrutinib were generally in patients who had relapsed on R‐CHOP. Although the data presented in the papers did not allow for statistical analysis of the survival data, the PFS between the different treatment regimens did not differ. This was true when looking at the range of survival data between papers, and when averaging the data in the different treatment regimens. Thus, the encouraging results of early trials of ibrutinib in MCL which resulted in ORR rates in the region of 70% [2, 68] did not result in long‐term survival benefits. This may be because the malignant cells of MCL patients quickly become resistant to ibrutinib by activating alternative pathways which promote their growth and survival [21, 69]. It is also important to take into consideration that ibrutinib was rarely used as a front‐line option, and the efficacy of all treatments for MCL decreases as the number of lines of treatment increases [66, 67, 70]. In the one study that did comment on the efficacy of ibrutinib when given as a front‐line treatment, the OS was 9.7 years, as compared with 41.1 m when given as second line [66]. The data suggest that ibrutinib given as the front‐line treatment might be a better option for elderly/unfit patients with MCL than R‐CHOP; however, this would need to be confirmed by further randomised control trials. Of note recent phase III studies adding rituximab to ibrutinib in CLL have shown that this does not improve the efficacy of the kinase treatment [71].

Furthermore, in the case of BR treatment, when analysing PFS data, it must be noted that patients treated with BR had advantageous prognostic features as compared to those treated with R‐CHOP and ibrutinib. These patients were younger, had less severe disease (fewer patients with stage III/IV disease) and a lower proportion were males; a fact which was highlighted in the data analysis of two of the studies comparing BR and R‐CHOP (Flinn et al 2014, [33]). Thus, although the data suggest that BR therapy was not inferior to R‐CHOP, it is important to take into consideration that this may not be the case because of the better demographics of the patients treated with BR. This is in line with a study completed in the United Kingdom after our data collection which found that the PFS of elderly/unfit patients treated in the United Kingdom with BR and R‐CHOP was not significantly different [72].

Another important issue to consider when deciding the best treatment option for MCL is the toxicity profile of the different regimens. This is particularly important when dealing with the elderly/unfit patient group who often have multiple comorbidities and are therefore less able to tolerate treatment side effects than their younger, and generally fitter counterparts. AEs were only reported in one study using R‐CHOP where they found a high number of haemic events ≥3 [65] as compared with other studies which found that severe AEs in elderly/unfit patients with MCL occur in < 5% of patients [2, 61]. The reasons for this discrepancy are unclear but highlight the importance of comparing data from different sources. As with R‐CHOP, the most common toxicities observed with ibrutinib and BR treatment were haemic. Patients treated with BR had more severe side effects than those treated with ibrutinib, although the demographics of the patient group were more favourable. By contrast, the number of patients who discontinued BR due to toxicity was higher than those who discontinued ibrutinib, these data were only included in one study of each treatment, and therefore warrant further validation in order to be definitively assessed. Taken together, the data indicate that the toxicity profile of BR is more severe than that of ibrutinib; and that both treatments have more severe toxicity profiles than R‐CHOP.

In conclusion, when taking into account the survival and toxicity profiles, R‐CHOP still remains the best treatment option for elderly/unfit patients with MCL. However, as with all treatments available, the duration of remission following front‐line treatment with R‐CHOP is approximately 2 years. Therefore, treatment of elderly/unfit patients with MCL still remains a clinically unmet need.

## CONFLICT OF INTEREST

The authors report no conflicts of interest.

## AUTHOR CONTRIBUTIONS

TA and MCP wrote the paper.

TA and MCP contributed to the analysis.

AT designed and performed the statistical analysis and outputs.

KJT designed the study and critically revised the paper.
